# Update to the dataset of cerebral ischemia in juvenile pigs with evoked potentials

**DOI:** 10.1038/s41597-021-01029-z

**Published:** 2021-09-23

**Authors:** Martin G. Frasch, Bernd Walter, Christoph Anders, Reinhard Bauer

**Affiliations:** 1 University of Washington School of Medicine, Center on Human Development and Disability, Seattle, WA USA; 2 Department of Spine Surgery and Neurotraumatology, SRH Waldklinikum, Gera, Germany; 3 Institute of Molecular Cell Biology, Jena University Hospital, Jena, Germany; 4 Clinic for Trauma, Hand and Reconstructive Surgery, Division of Motor Research, Pathophysiology and Biomechanics, Jena University Hospital, Jena, Germany

**Keywords:** Neurophysiology, Stroke

## Abstract

We expand from a spontaneous to an evoked potentials (EP) data set of brain electrical activities as electrocorticogram (ECoG) and electrothalamogram (EThG) in juvenile pig under various sedation, ischemia and recovery states. This EP data set includes three stimulation paradigms: auditory (AEP, 40 and 2000 Hz), sensory (SEP, left and right maxillary nerve) and high-frequency oscillations (HFO) SEP. This permits derivation of electroencephalogram (EEG) biomarkers of corticothalamic communication under these conditions. The data set is presented in full band sampled at 2000 Hz. We provide technical validation of the evoked responses for the states of sedation, ischemia and recovery. This extended data set now permits mutual inferences between spontaneous and evoked activities across the recorded modalities. Future studies on the dataset may contribute to the development of new brain monitoring technologies, which will facilitate the prevention of neurological injuries.

## Abstract (summary)

**Table:** 

Measurement(s)	Abnormal auditory evoked potentials • Abnormality of somatosensory evoked potentials • high frequency oscillations • brain activity
Technology Type(s)	Electrocorticography • electrothalamography
Factor Type(s)	anesthesia • analgosedation • sedation • cerebral ischemia
Sample Characteristic - Organism	Sus scrofa

Machine-accessible metadata file describing the reported data: 10.6084/m9.figshare.16462875

## Background & Summary

This extension dataset adds the dimension of *evoked* potentials (EPs) to the recently published^1^ dataset containing *spontaneous* ten-channel electrocorticogram (ECoG) and electrothalamogram (EThG) activities, accompanied by the data on cerebral blood flow^2^, cardiovascular dynamics and metabolites. Specifically, we present the recordings of the sensorimotor and auditory EPs (SEPs, AEPs) during the same conditions of several sedation states, followed by gradual and controlled cerebral ischemia and 60 minutes of recovery as presented for the spontaneous activity^1^.

The model remains to be that of juvenile pigs where we originally introduced the basic stereotaxic approach to chronically recording EThG and quantifying the effects of isoflurane and fentanyl sedation on the brain electrical activity from a ten-channel ECoG, EThG, and the cerebral blood flow^2^, followed by the characterization of the effects of gradual propofol sedation on these parameters^3^.

As for the study of spontaneous brain electrical and cardiovascular activities, for the study of EPs the choice of this animal model is also dictated by its excellent amenability to complex stereotactic chronic instrumentation, including the well-controlled application of SEPs via snout electrodes and AEP via in-ear headphones, prolonged stimulation required for high-frequency oscillations (HFO) SEPs^4,5^, studies of sedation and clinically relevant patterns of hypoxic/ischemic injury in a relatively large and gyrencephalic brain^2^.

We hope this dataset will contribute to the exciting area of ongoing research into the physiology of SEPs, HFO SEPs^6^, and mid-latency AEPs^7,8^ as manifestations of thalamocortical or intracortical communication that can be harnessed as biomarkers of sedation, cerebral ischemia and neuronal recovery.

Furthermore, the herein offered dataset supports translation of experimental animal findings to clinical applications of EPs during ischemic events. Recently, a seminal clinical study revealed the usefulness of HFO SEPs to support decision-making after global brain ischemia due to cardiac arrest and resuscitation after severe brain injury^9^. Endisch *et al*. verified that analysis of high-frequency SEP components may improve prognostication of the absence of severe hypoxic encephalopathy following cardiac arrest.

For the technical validation, we present the complete raw and processed SEP, HFO SEP and AEP datasets as well as a representative analysis.

We hope the present EP dataset will contribute to the development of new brain monitoring technologies, which will facilitate the prevention of neurological disorders.

All experiments were carried out in accordance with the European Communities Council Directive 86/609/EEC for animal care and use. The Animal Research Committee of the Thuringian State government approved laboratory animal protocols.

## Methods

### Instrumentation

#### General instrumentation

The protocol was approved by the Committee of Animal Care and Use of the Thuringian State Government

(Germany). We surgically instrumented eleven mixed breed female pigs (7-weeks-old, 14.9 ± 1.2 kg body weight). The detailed approach has been published^1,2^.

#### Instrumentation of the head

**Fig. 1:**
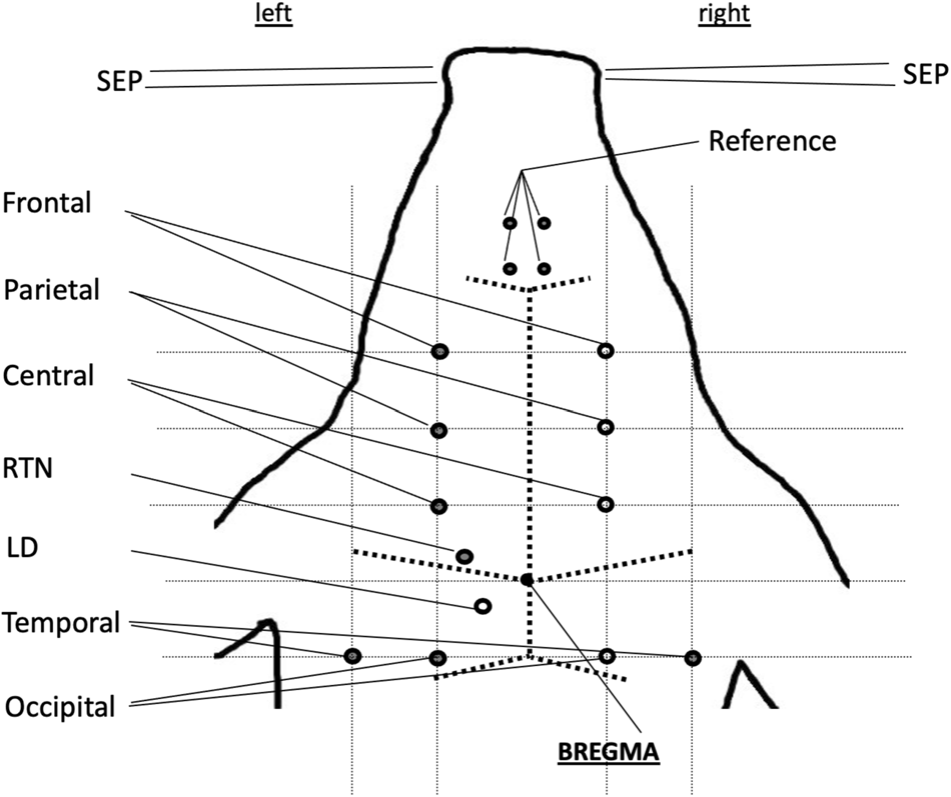
Instrumentation of pig for recording ECoG and EThG. Note that EThG was recorded from the left thalamus’ RTN and LD nucleus.

The head instrumentation procedure is identical to the previously reported approach (Table 1, Fig. 1)^1^.

**Table 1:** Stereotactic coordinates* (reproduced with modifications from^1^).

Position	Lateral of sagittal suture	Anterior (a)/Posterior (p) of bregma	Depth from the dura	Equivalent according to 10/20 single plane projection of the head^23^
Frontal ECoG, Ch. 1 & 2 (left & right)	12 mm	a 30 mm		Fp1, Fp2
Parietal ECoG, Ch 3 (left^#^ & right)	12 mm	a 20 mm		F3^#^, F4
Central ECoG, Ch. 4 & 5 (left & right)^$^	12 mm	a 10 mm		C3, C4
Temporal ECoG, Ch. 6 & 7 (left & right)	24 mm	p 10 mm		T5 = P7, T6 = P8
Occipital ECoG, Ch. 8 & 9 (left & right)	12 mm	p 10 mm		P3, P4
EThG, Ch. 10 (RTN = Nucl. reticularis thalami)	9 mm	a 2 mm	24 mm	
EThG, Ch. 11	5 mm	p 2 mm	20 mm	

(LD = Nucl. dorsolateralis thalami)

*Reference: Nz

^#^excluded from the dataset due to poor signal quality.

^$^channels of particular interest for HFO SEP study^6^.

In addition, the following steps were performed to enable SEP and AEP studies.

SEP electrodes were installed bilaterally on the outer edges of the pig snout to stimulate maxillary nerves.

For AEP studies, the animal’s ears were first freed from the stereotactic apparatus. Then, a miniature in-ear headset was inserted on each side (Sennheiser MX 300).

### Description of experimental stages

**Fig. 2:**
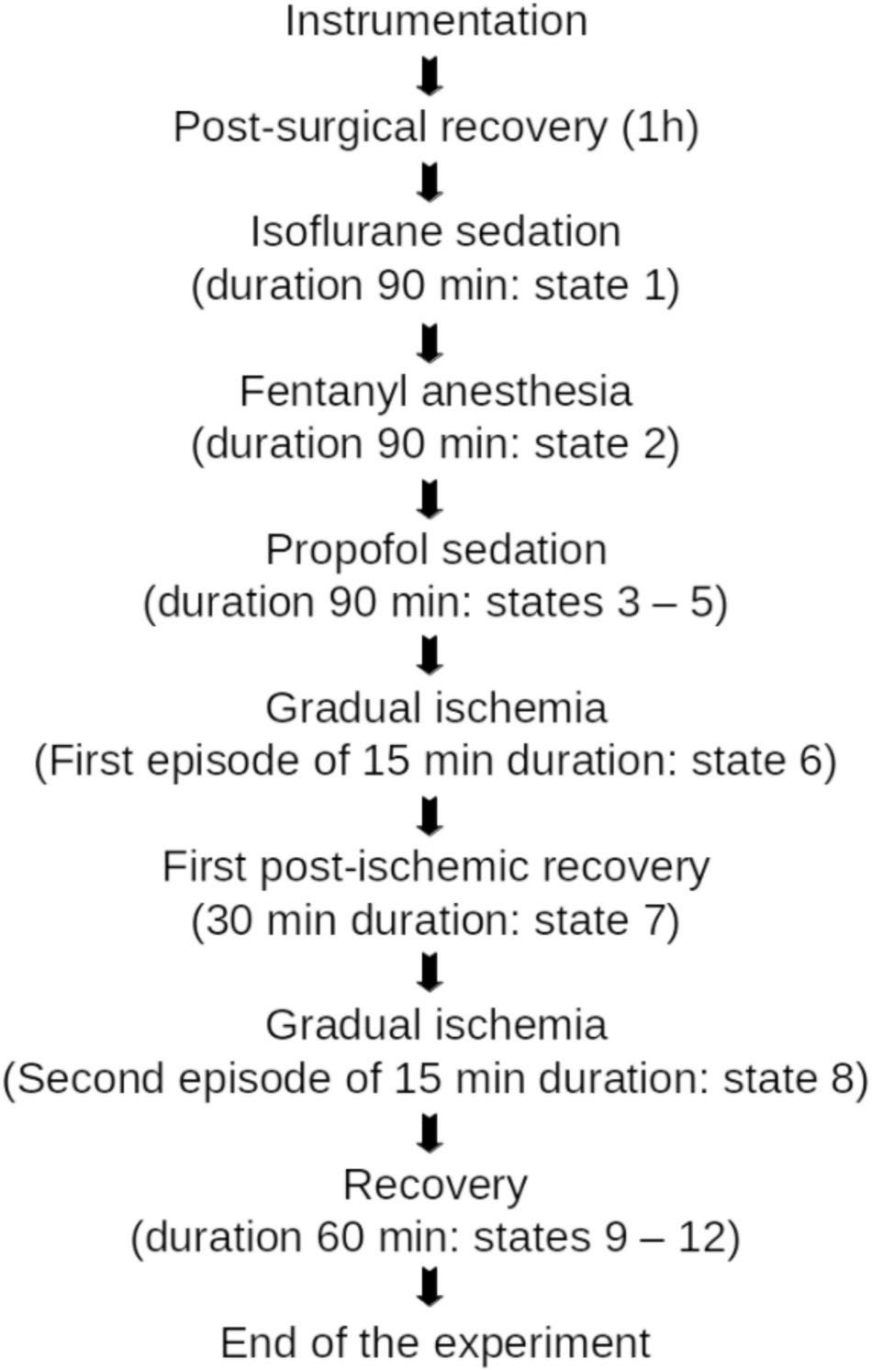
Experimental protocol of pig model of sedation and gradual ischemia.

The experimental protocol is summarized in Fig. 2 and has been presented in^1^.

Briefly, during the initial period of post-surgical recovery, the intra-surgical analgosedation was maintained. After that period, the pigs were allowed 90 minutes to stabilize (**State 1**). During the entire experimental period, electrocorticogram (ECoG) and electrothalamogram (EThG) were recorded continuously. Following State 1, isoflurane in N_2_O and O_2_ were discontinued and ventilation with 100% O_2_ was performed for 5 minutes. **State 2a** began, characterized by an intravenous bolus injection of fentanyl (0,015 mg/kg b.w.), followed by **State 2b -** a continuous iv infusion of fentanyl (0.015 mg/kg b.w./h) for 90 minutes. **States 3–5** represented propofol anesthesia of various degrees and were performed in six animals. To induce **State 3**, deep anesthesia, first, we determined the individual doses of propofol required for the maintenance of deep anesthesia under continuous control of mean ABP (MABP). Next, propofol was infused intravenously (0.9 mg/kg BW/min for ~ 7 min) until a burst suppression pattern (BSP) appeared in the ECoG. The depth of anesthesia was subsequently maintained for 25 minutes via propofol administration (~0.35 mg/kg b.w./min). Next, to transition to moderate propofol anesthesia, 30% of the propofol dose required for BSP induction was continuously administered over the course of 90 minutes. About ten minutes after the onset of the moderate anesthesia period, the first measurement was performed **(State 4)**. Following 60 min, **State 5** measurement was performed.

The gradual cerebral ischemia was induced as follows^10^. First, the cisterna magna was punctured by a lumbar puncture needle fixed in place by dental acrylic resin for elective artificial cerebrospinal fluid infusion/withdrawal allowing the control of intracranial pressure (ICP). Subsequently, we installed a plastic-coated cerclage on the pulmonary trunk to enable mechanical control of the pulmonary trunk diameter. This method allowed us to control cardiac output and adjust the MABP to about 90 mmHg. The ICP increase was achieved by the infusion of artificial cerebrospinal fluid (warmed to 37 °C) into the subarachnoid space via the punctured cisterna magna. We calculated the cerebral perfusion pressure (CPP) as the difference between MABP and the ICP. By curbing MABP rise and elevating ICP, we were able to decrease the CPP to 25 mmHg. The Cushing response during severe brain ischemia was prevented by the appropriate curbing of the pulmonary trunk diameter. Finally, to stop cerebral ischemia we opened the cerclage completely and withdrew the artificial cerebrospinal fluid reaching an ICP < 10 mmHg.

The states of gradual ischemia were maintained for 15 min twice (**States 6 and 8**), interceded by a recovery state (**State 7**) lasting 30 minutes in all animals (P746 - P794) except P739 where it lasted 15 minutes. This was followed by 60 min of recovery (**States 9–12**).

We confirmed the stereotaxic position of the electrodes visually and histologically on the fixed brains, as previously reported^2^. We report the validation of the animal-specific position of the thalamic electrodes in Table 2. The correct position was confirmed for 18 out of 22 thalamic electrodes. The incorrect placements amounted to 2 mm off target and the brain regions were documented (Table 2). In one case, both electrodes were 2 mm outside the target region. In two further animals, the electrode tips of either RTN or LD electrodes were outside the target region. 1 mm off-target was observed in the case of two electrodes and allowed (i.e., deemed “ok”). We noted this also explicitly in Table 2.

**Table 2:** Validation of localization of the thalamic electrodes.

ID	Age, wk	Bodyweight, kg	Brain weight, g	Electrode position RTN/LD	Notes
728	7	14	70.17	ok/ok	
737	7	15	72.43	ok/ok	RTN electrode 1 mm lateral, WM*
738	7	18	65.55	ok/ok	
739	7	13.5	73.94	ok/f	ventral
743	6	15	69.84	f/f	RTN: 2 mm lateral, inf. temp. gyrus; LD: 1–2 mm lateral, WM*
746	7	14	71.48	ok/ok	
749	7	15	72.97	f/ok	RTN: 2 mm lateral, WM*
752	7	15	77.86	ok/ok	RTN: 1 mm lateral, WM*, assumed “OK” (caution with data)
753	7	15	71.4	ok/ok	
791	8	16	71.4	ok/ok	
794	9	17	71.4	ok/ok	
**Mean**	7.2	15.2	71.7	9 (11)/9 (11)	
**SD**	0.8	1.3	3.0		

^*^white matter; wk, week.

On the onset of each state, the evoked potentials were administered in the following order:

1. SEP left
2. SEP right
3. AEP 40 Hz
4. AEP 2000 Hz
5. HFO SEP required up to 16 minutes of stimulation duration and, hence, were not performed for each state due to limitations in the time allotted for each state.

Table 3 reviews which recordings are available for each state.

**Table 3:** Review of experimental stages and the respective available data sets*.

State #	Experimental stage	EP measurements	Available data sets
state1	Isoflurane	SEP, AEP	P_728, P_737, P_738, P_739, P_743, P_746, P_749, P_752, P_753, P_791, P_794
		HFO SEP	P_743, P_746, P_749, P_752, P_753, P_791, P_794
state2	Fentanyl	SEP, AEP	P_728, P_737, P_738, P_739, P_743, P_746, P_749, P_752, P_753, P_791, P_794
		HFO SEP	P_739, P_749, P_753, P_791, P_794
state2.5	90 min post-fentanyl	SEP, AEP	P_739, P_749, P_753, P_791,
state3	Propofol	SEP, AEP	P_728, P_737, P_738, P_743, P_746, P_752
state4	Moderate sedation - immediate measurement	SEP, AEP	P_728, P_737, P_738, P_743, P_746, P_752
		HFO SEP	P_737, P_738, P_743, P_746, P_752
state5	60 min post-moderate sedation	SEP, AEP	P_737, P_738, P_743, P_746, P_752
state5.5	Fentanyl directly pre-ischemia	SEP, AEP	P_794
state6	1st ischemic phase: gradual ischemia	SEP, AEP	P_728, P_737, P_738, P_739, P_743, P_746, P_749*, P_752, P_753, P_791, P_794
state7a	15 min recovery post-ischemia (first period)	SEP, AEP	P_739, P_746, P_749, P_752, P_753, P_791, P_794
state8	2nd ischemic phase: gradual ischemia	SEP, AEP	P_739, P_746, P_749, P_752, P_753, P_791, P_794
state9	15 min recovery post-ischemia	SEP, AEP	P_737, P_743 (after single ischemia period); P_739, P_743, P_746, P_749, P_752, P_753, P_791, P_794 (after second ischemia period)
state10	30 min recovery post-ischemia	SEP, AEP	P_746, P_749, P_752, P_753, P_791, P_794
state11	45 min recovery post-ischemia	SEP, AEP	P_737, P_739, P_743, P_749, P_752, P_753, P_791, P_794
state12	60 min recovery post-ischemia	SEP, AEP	P_737, P_743, P_746, P_749, P_752, P_753, P_791, P_794

^*^Details including any artifacts observed, for each file and for each channel, are provided in the auxiliary spreadsheet on Figshare^13,24^.

#### Data acquisition and analyses

The approach is identical to what we previously reported^1^.

In addition, the following methods were used to trigger and record SEP and AEP.

As reported in^2^, SEPs were induced by bipolar application of constant current rectangular impulses (70 ms, 5 mA applied at 1 pulse/s (HES-Stimulator T, Fa. Hugo Sachs Elektronik, Hugstetten, Germany), for 2 minutes at every side, respectively. The recordings were sampled at 2 kHz and averaged across 100 sweeps.

AEPs were induced by applying auditory stimuli first at 40 Hz and then at 2000 Hz. The auditory stimuli were administered at 90 dB, 150 µs DC impulses, 40 ms duration followed by 5/s frequency with filter bandpass of 15–1500 Hz. The recording electrodes were placed bilaterally on the mastoid; the reference was placed on the vertex. These stimulation parameters were generated using a compiled version of the custom Matlab script provided by Dr. Akeroyd’s lab (available for download at the referenced permanent URL)^7,11,12^.

HFO SEPs were induced by bipolar application of constant current rectangular impulses (70 ms, 5 mA applied (HES-Stimulator T, Fa. Hugo Sachs Elektronik, Hugstetten, Germany), at a frequency of 5 Hz or 9 Hz for 16 min or 10 minutes at every side, respectively. Raw data files were band-pass filtered between 400 Hz and 800 Hz with an FIR digital filter according to^4^ and averaged.

Raw files with constant current rectangular impulse stimulation displayed frequently short-term impulse-synchronized artifacts (duration 1 ms at the stimulus time point) with detectable alteration on FIR digital filter effects. Therefore, for each ECoG and EThG channel, we removed 4 ms before and after every stimulus and replaced the missing data with a moving average between the previous and subsequent values.

### Signal analysis methodology

Evoked potentials were calculated using the EP plugin of Watisa® software. Therefore, recorded stimulus signals were used for event triggering. Subsequently, signal segments of 100 ms duration, starting at the trigger time point, were averaged and stored. Finally, respective EPs determined under identical experimental conditions were averaged as grand mean and used for further analysis.

## Data Records

**Fig. 3:**
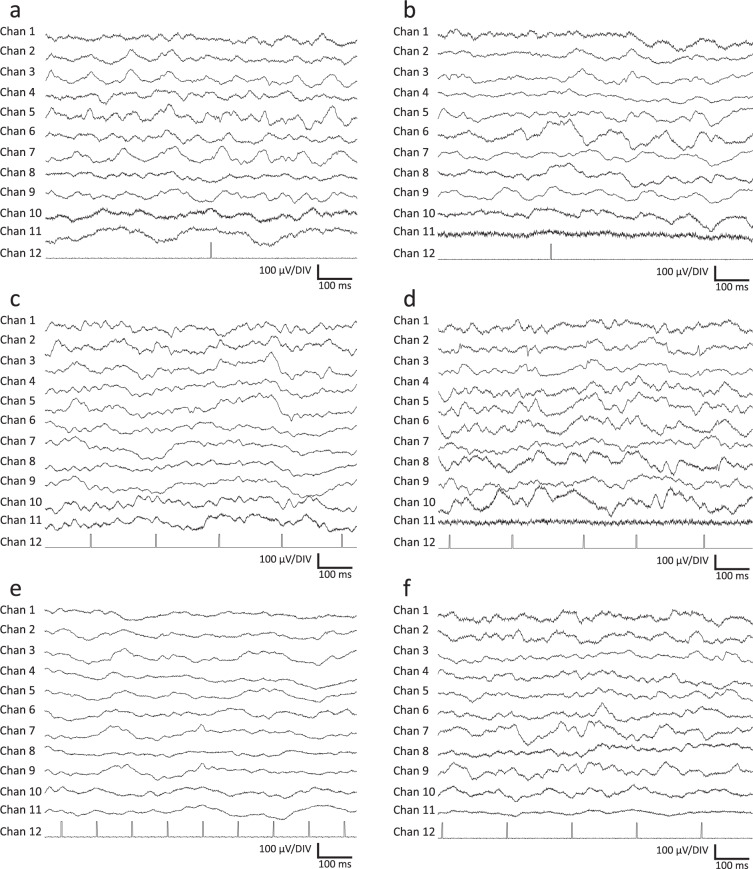
Representative raw recording during data acquisition in Watisa® software (State 1). (**a**) left-side sensorimotor EP stimulation, (**b**) right-side sensorimotor EP stimulation, (**c**) auditory EP stimulation with 40 Hz, (**d**) auditory EP stimulation with 2 kHz, (**e**) sensorimotor EP stimulation with 9 Hz repetition frequency for high-frequency oscillation SEP analysis, (**f**) sensorimotor EP stimulation with 5 Hz repetition frequency for high-frequency oscillation SEP analysis. Twelve channels are given at a sampling rate of 2000 Hz (Chan 1, frontal left; Chan 2, frontal right; Chan 3, parietal right; Chan 4, central left; Chan 5, central right; Chan 6, temporal left; Chan 7, temporal right; Chan 8, occipital left; Chan 9, occipital right; Chan 10, RTN nucleus (left); Chan 11, LD nucleus (left); Chan 12, EP stimulus channel).

A representative recording is shown in Fig. 3.

The data structure and annotation are as follows. 12 channels are given at a sampling rate of 2000 Hz and a recording duration of about 300 s per state each containing the following channels.

Channels 1–9     ECoG, frontal, parietal, central, temporal, and occipital regions

                                (left parietal ECoG was removed due to artifacts, as before)

Channels 10, 11 EThG from RTN and LD nucleus

Channel 12          SEP or AEP triggers, respectively

Figure 1 and Table 1 show the electrode arrangement. ECoG channel 3 was of poor quality in most instances due to intermittent technical problems (random faults) and an amplifier breakdown. We hence removed this channel from the dataset presented. That means that the left parietal ECoG (channel 3) is not included in the dataset.

All data have been deposited on Figshare in BIDS format^13^.

The grand means and the underlying individual averaged EP data are available on Figshare and are named and structured as follows:

- “AEP-40Hz”
- “AEP-2000Hz”
- “SEP-left”
- “SEP-right”
- “HFO-SEP”

○(“HFO-SEP_full_length.xlsx” are HFO SEP grand means where the entire HFO SEP segment was computed and grand means were calculated. The result is then rendered graphically.

○“HFO-SEP_5–50 ms.xlsx” are HFO SEPs where the above original individual HFO SEP data were cut 0.5–4.5 ms and from 50.5 ms until the end. The result is then rendered graphically. This version removes the initial ~5 seconds 400–800 Hz bandpass FIR swinging artifact on the left side and the higher-order latencies on the right side. The latter may be of interest for future studies on this dataset.

The raw EP data is deposited archived (to retain the file structure) and in the European Data Format (EDF).

“Roadmap for file assessment” file is a spreadsheet that reviews the quality of each recording as we did for the previous dataset.

A total of 11 channels are rendered for the states 1–12 using the same Y-axis settings to ensure the evolution of evoked potentials is captured.

The accompanying file with the table “Number of channels by state and condition” informs how many channels were averaged per state.

## Technical Validation

We present the approach and findings of SEPs and AEPs as well HFO SEPs in the ECoG and EThG under the states of sedation, ischemia and recovery.

These data can be used to corroborate and enhance the recently published studies in brain-injured human subjects where the patterns of SEPs were investigated in conjunction with postmortem histopathological findings including thalamic injury and with related (patho-) physiological parameters^14^.

**Fig. 4:**
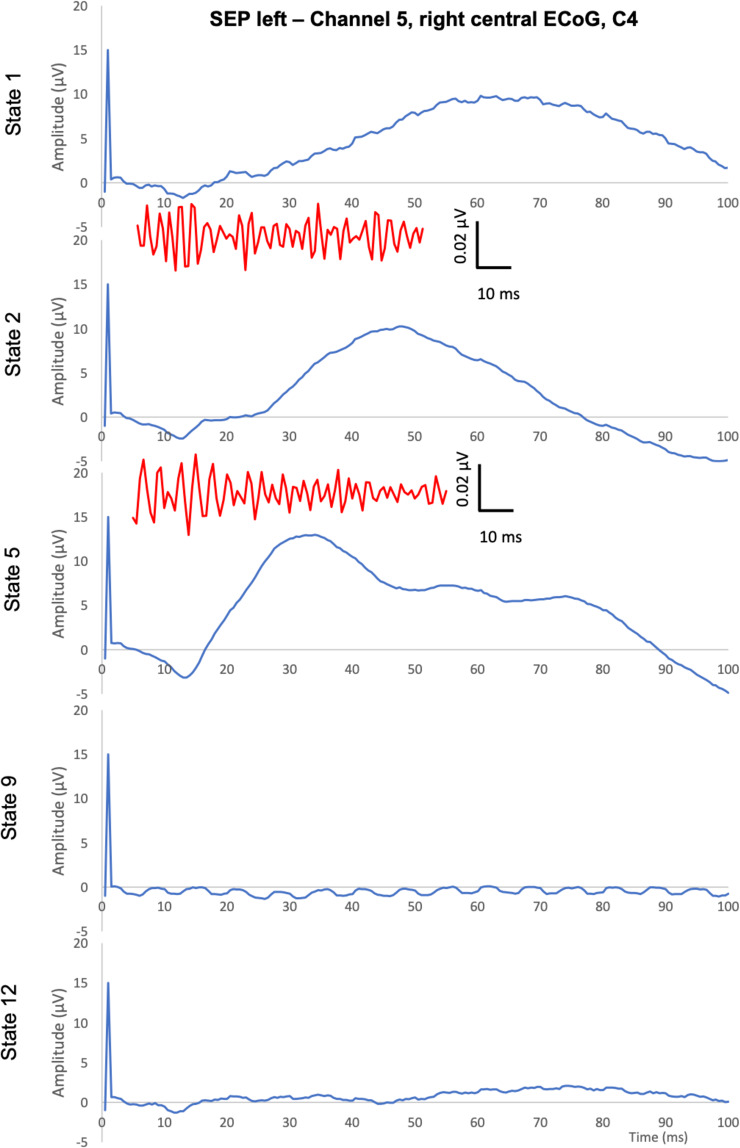
Representative findings of left SEPs and HFO SEPs (provided between 5 and 50 ms post-stimulus and sharing the X-axis scale with SEPs). Left SEPs are shown in the respective contralateral ECoG channel 5 along with the corresponding HFO responses (in red).

**Fig. 5:**
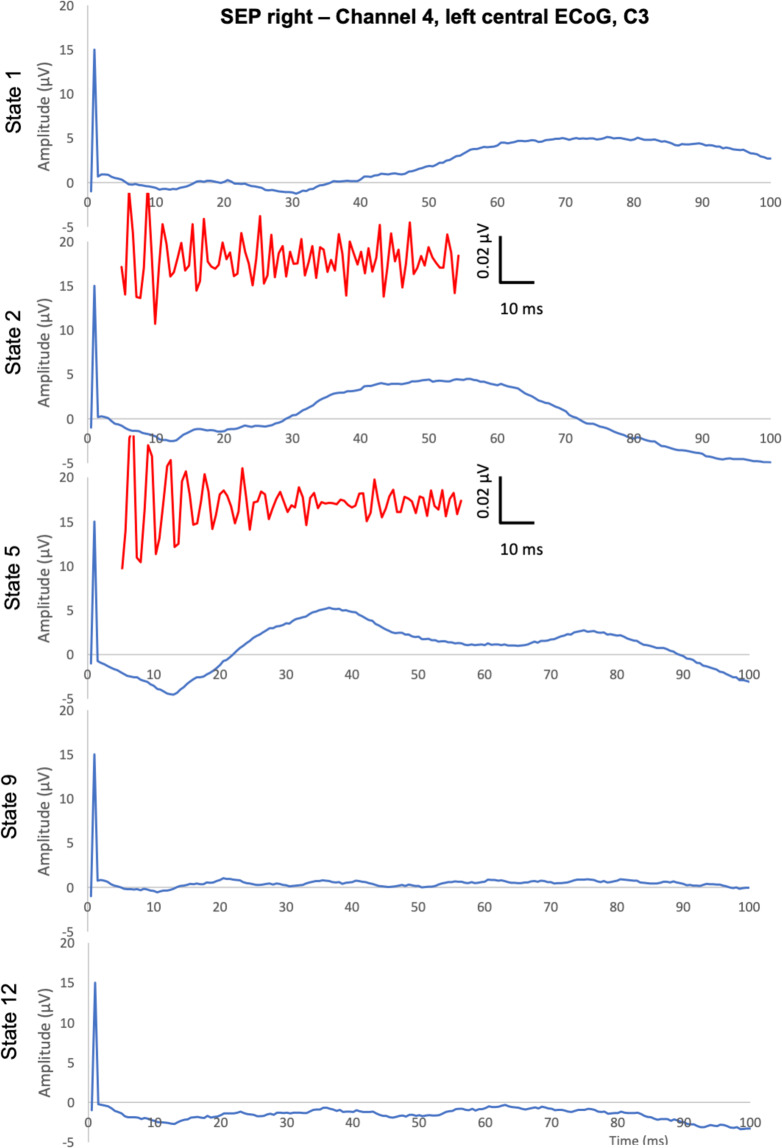
Representative findings of right SEPs and HFO SEPs (provided between 5 and 50 ms post-stimulus and sharing the X-axis scale with SEPs). Right SEPs are shown in the respective contralateral ECoG channel 4 along with the corresponding HFO responses (in red).

**Fig. 6:**
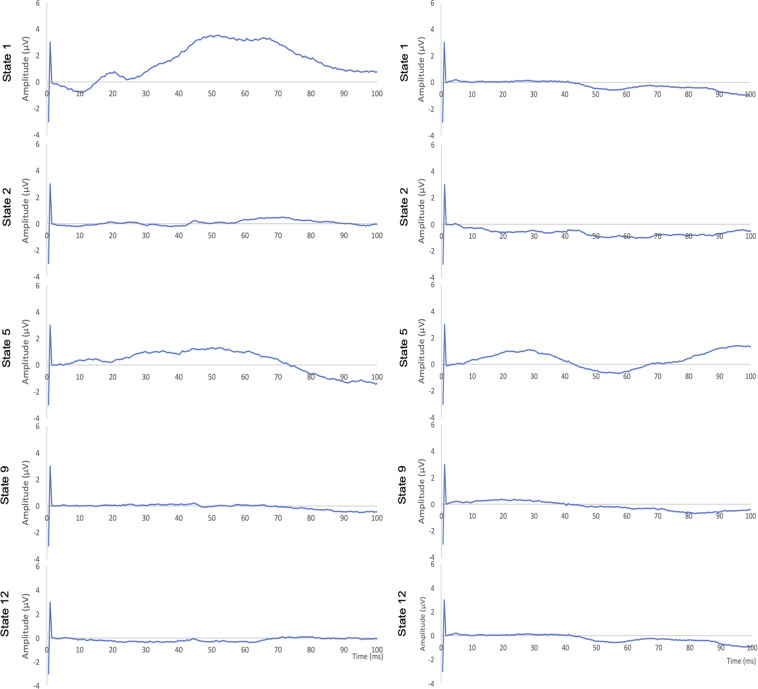
Representative findings of AEPs. Central ECoG (right, C4). Left: 40 Hz; Right: 2000 Hz.

**Fig. 7:**
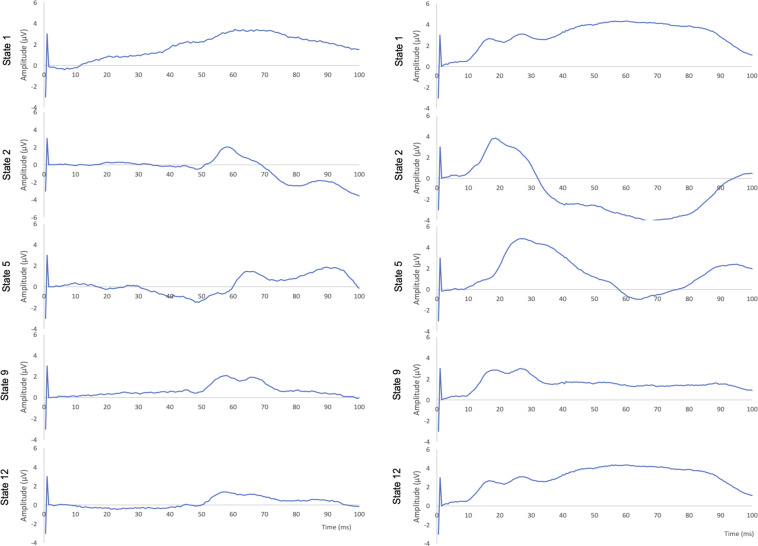
Representative findings of AEPs. Temporal ECoG (right, P8). Left: 40 Hz; Right: 2000 Hz.

The SEPs and AEPs are presented as grand means. The complete results are available in the accompanying repository on Figshare^13^. Here we provide the representative findings for sedation, ischemia and recovery states as the grand means (Figs. 4–7).

Clearly, the herein used methods for data analysis represent merely a starting point to illustrate data quality and usefulness for future in-depth analyses. The presented data set – in combination with already published cerebrovascular data (including CMRO_2_ and regional CBF)^1^ – enables a systematic and comprehensive analysis of SEPs and AEPs as well HFO SEPs in the ECoG and EThG. Data sets are classified into different state-dependent traits: (i) different qualities of sedation (isoflurane; propofol), (ii) pure analgesia (fentanyl), (iii) repeated gradual brain ischemia, and (iv) different periods of post-ischemic recovery. Note that the complete data set offers the possibility to verify the effects of combined moderate propofol sedation/fentanyl analgesia or pure fentanyl analgesia on electrophysiological responses during gradual brain ischemia periods and post-ischemic recovery. Such approaches are likely to deliver deepened functional insights to improve the prognostication value of clinical studies where cohort SEPs are correlated with histopathological findings after transient global ischemia^15^.

For SEPs, we present the first 100 ms which corresponds to the timeframe of the ipsilaterally elicitable early and contralaterally triggered mid-latency SEPs (Figs. 4 and 5). Mid-latency SEPs (e.g., N20, P20) allow simultaneous study of the temporally overlapping HFO SEPs^5,6,16^. Consequently, for representative purposes here, we present the Channels 4 and 5 (central ECoG corresponding to C3 and C4) respectively contralateral to the stimulation site. SEP and the corresponding HFO SEP are shown. Note that HFO 5 Hz stimulation only (and not 9 Hz) appears to show correspondence to the mid-latency oscillations of SEP. We observe N20 and P20 components and their HFO counterparts for the first two states of isoflurane and fentanyl sedation, respectively. As noted, not all HFO SEP recordings could be obtained due to the limitations of the required stimulation duration. In the conventional SEPs, the clear modulatory impact of the different sedation modes and the abolition of the response following cerebral ischemia are apparent.

For AEPs, we also focused on the first 100 ms which encompass the brainstem (V), mid-latency (MLAEP, Na, Pa, Nb, Pb) thalamically generated responses, early cortical AEPs (EAEP, from primary auditory cortex) and late cortical responses (LAEP, from frontal cortex and the association areas) (Figs. 6 and 7). Overall, our findings agree with the literature^7^. Note the mid-latency peak under isoflurane (state 1) which is abolished under fentanyl (state 2), slightly restored under moderate propofol sedation (state 5) and again extinguished 15 and 60 minutes after cerebral ischemia (states 9 and 12 respectively). The temporal ECoG signal shows a more dynamic response overall than central ECoG, as can be expected, with some frequency specificity and partial (40 Hz) or complete recovery (2000 Hz) 60 minutes post-ischemia.

The grand means represent the averages of all evoked potential measurements per ECoG/EThG channel per state.

## Usage Notes

We recognize that not all EP, in particular HFO SEP, measurements are available for all states and animals. The duration of time intervals between the experimental states left sometimes little room for some of the measurements and we prioritized obtaining the conventional SEP and AEP data over the longer-lasting HFO SEPs. We have to consider that there is no information available on the voltage noise density of the DC amplifier (DC-EEG-AMPLIFIER, Schwind Medizintechnik, Erlangen, Germany) used for ECoG and EThG recordings. It should be taken into account that lowering SEP machine-related intrinsic noise levels improves signal-to-noise ratio and is crucial in future animal and human studies on HFO SEPs^17^.

We inventoried the dataset in the roadmap spreadsheet, as part of the Figshare repository, to help navigate the data and design optimal secondary investigations on the data.

As we reported before^1^, the present dataset has been acquired at a 2,000 Hz sampling rate and is hence amenable to studies of the properties of AEPs and SEPs in relation to the HFOs under conditions of various sedation regimes^18–20^. Consequently, thanks to concomitant EThG recordings, it is possible to relate the ECoG patterns of spontaneous HFOs to their thalamic contributions in EThG and their representation in the evoked responses.

## Data Availability

EEGLAB has been used which is available as open-source^21^. ERPlab plug-in for EEGLAB can be used as a freely available solution for further EP analysis^22^. No proprietary code has been deployed in this study except for WATISA (GJB Datentechnik GmbH, Ilmenau, Germany).

## References

[1] Frasch MG, Walter B, Herry CL, Bauer R (2021). Multimodal pathophysiological dataset of gradual cerebral ischemia in a cohort of juvenile pigs. Sci Data.

[2] Frasch MG (2006). Stereotactic approach and electrophysiological characterization of thalamic reticular and dorsolateral nuclei of the juvenile pig. Acta Neurobiol. Exp..

[3] Frasch MG (2007). Detecting the signature of reticulothalamocortical communication in cerebrocortical electrical activity. Clin. Neurophysiol..

[4] Ikeda H, Leyba L, Bartolo A, Wang Y, Okada YC (2002). Synchronized spikes of thalamocortical axonal terminals and cortical neurons are detectable outside the pig brain with MEG. J. Neurophysiol..

[5] Ikeda H, Wang Y, Okada YC (2005). Origins of the somatic N20 and high-frequency oscillations evoked by trigeminal stimulation in the piglets. Clin. Neurophysiol..

[6] Ozaki I, Hashimoto I (2011). Exploring the physiology and function of high-frequency oscillations (HFOs) from the somatosensory cortex. Clin. Neurophysiol..

[7] Martoft L (2001). Middle-latency auditory evoked potentials during induction of thiopentone anaesthesia in pigs. Lab. Anim..

[8] Iselin-Chaves IA, E Moalem HE, Gan TJ, Ginsberg B, Glass PS (2000). Changes in the auditory evoked potentials and the bispectral index following propofol or propofol and alfentanil. Anesthesiology.

[9] Endisch C (2016). Cortical somatosensory evoked high-frequency (600Hz) oscillations predict absence of severe hypoxic encephalopathy after resuscitation. Clinical Neurophysiology.

[10] Walter B (2010). Age-dependent effects of gradual decreases in cerebral perfusion pressure on the neurochemical response in swine. Intensive Care Med..

[11] Akeroyd MA (2017). University Of Nottingham.

[12] Akeroyd MA (2007). The binaural performance of a cross-talk cancellation system with matched or mismatched setup and playback acoustics. The Journal of the Acoustical Society of America.

[13] Frasch M, Walter B, Anders C, Bauer R (2021). figshare.

[14] van Putten MJAM (2019). Postmortem histopathology of electroencephalography and evoked potentials in postanoxic coma. Resuscitation.

[15] Endisch C (2020). Hypoxic-Ischemic Encephalopathy Evaluated by Brain Autopsy and Neuroprognostication After Cardiac Arrest. JAMA Neurol..

[16] Niedermeyer, E. & Da Silva, F. L. *Electroencephalography*. vol. 4th (Lippincott Williams & Wilkins, 1999).

[17] Fedele T (2017). Intraoperative subdural low-noise EEG recording of the high frequency oscillation in the somatosensory evoked potential. Clin. Neurophysiol..

[18] Thomschewski A, Hincapié A-S, Frauscher B (2019). Localization of the Epileptogenic Zone Using High Frequency Oscillations. Front. Neurol..

[19] Srejic LR, Valiante TA, Aarts MM, Hutchison WD (2013). High-frequency cortical activity associated with postischemic epileptiform discharges in an *in vivo* rat focal stroke model. J. Neurosurg..

[20] Bragin A (2002). Interictal high-frequency oscillations (80-500 Hz) in the human epileptic brain: entorhinal cortex. Ann. Neurol..

[21] Delorme A, Makeig S (2004). EEGLAB: an open source toolbox for analysis of single-trial EEG dynamics including independent component analysis. J. Neurosci. Methods.

[22] Lopez-Calderon J, Luck SJ (2014). ERPLAB: an open-source toolbox for the analysis of event-related potentials. Front. Hum. Neurosci..

[23] Klem, G. H., Jasper, H. H. & Elger, C. The ten±twenty electrode system of the International Federation. 10590970

[24] Frasch M, Bauer R (2019). figshare.

